# Therapeutic Management of Giant Uterine Fibroid: A Case Report

**DOI:** 10.7759/cureus.48169

**Published:** 2023-11-02

**Authors:** Mehak Rout, Apoorva Dave, Sanket S Bakshi

**Affiliations:** 1 Department of Obstetrics and Gynaecology, Jawaharlal Nehru Medical College, Datta Meghe Institute of Higher Education and Research, Wardha, IND; 2 Department of Medicine, Jawaharlal Nehru Medical College, Datta Meghe Institute of Higher Education and Research, Wardha, IND

**Keywords:** imaging, menstruation, interventional radiology, large fibroid, uterine artery embolization

## Abstract

The most common benign tumor that affects female reproductive system is a uterine fibroid or leiomyomas, especially during their reproductive years. The prevalence is around 20-40% women in the age group of 14-45 years. The following case report proffers the diagnosis and management of a female diagnosed with uterine fibroids. In this case a 45-year-old woman presented to the tertiary rural hospital with an unusually large distended abdomen which on examination and imaging revealed a big fibroid growing inside the uterus. The case highlights the significance of a collaborative approach involving gynecologists, interventional radiologists, and surgeons. Their combined expertise provides patients with various treatment options: medical management, minimally invasive procedures, and surgical interventions. During the decision-making process, factors such as the patient's age, desire for fertility preservation, and impact of fibroids on her quality of life are taken into account. This particular case showcases successful pre-hysterectomy uterine artery embolization of large uterine leiomyoma while emphasizing the importance of personalized care and shared decision making for optimal patient outcomes.

## Introduction

The prevalence of uterine fibroids or leiomyoma, which is a common benign tumor, is higher in women of reproductive age i.e. 14-45 years. The prevalence is almost as high as 20-40% in women of said age [1]. These tumors are well-known for being notoriously asymptomatic and most of the time found unintentionally during clinical examinations or during imaging [2]. When symptomatic it presents with excessive or unusual menstruation, frequent and urgent urination, bowel dysfunction, lower back pain, increased pelvic pressure, urine retention, dyspareunia and constipation. The first imaging modality that is preferred is ultrasonography [3]. A lot of the information about the connection between symptoms and the presence of fibroids is based on clear studies that looked at how myomectomy affected presenting symptoms [4]. Even though they are common amongst women of reproductive age, such a presentation of uterine fibroid in a tertiary clinical setting was rather unexpected. The initial presentation, diagnostic problems, and multidisciplinary therapeutic strategy used in a rural tertiary care hospital for a patient with a huge uterine fibroid are all covered in this case report. This particular instance shows not only the clinical nuances associated with gigantic fibroids but also the significance of innovative management approaches in healthcare settings with limited resources. In this report, we provide the clinical information, radiological results, surgical procedure, and postoperative treatment catered to this patient's particular need. The example that is being discussed is evidence of the importance of flexible healthcare strategies in treating complicated gynecological disorders, especially in remote areas where access to cutting-edge healthcare facilities may be restricted.

## Case presentation

**Figure 1:**
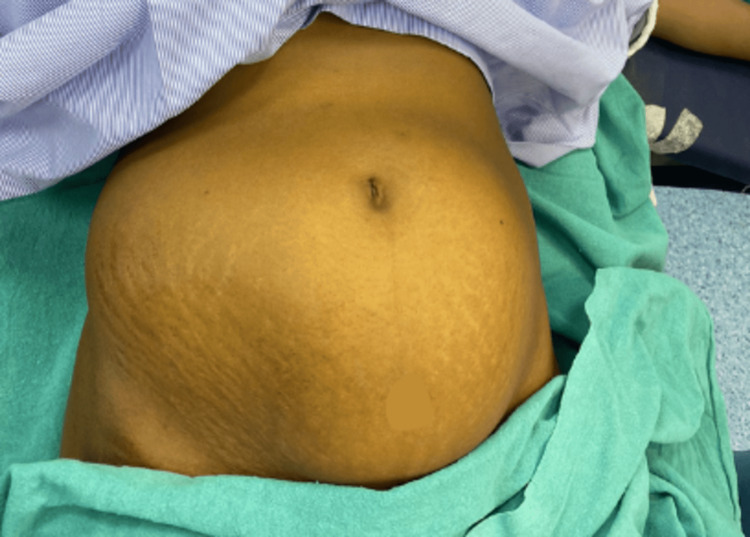
Distended abdomen found on general examination

A 45-year-old multiparous woman, para two, live births two presented at a rural tertiary care center with complaints of excessive menstrual bleeding and a palpable abdominal mass for the last three years. The mass consecutively increased in size causing abdominal distention and on physical examination her abdomen was found to be distended, measuring approximately 28 weeks of gravid uterus as seen in Figure 1. On examination of the pelvis a significantly enlarged uterus was appreciated. On palpation the uterus was firm to hard in consistency given the large size of the fibroid inside it, this also affected mobility since large fibroids can distort the shape of the uterus into and irregular mass and make it fixed into an abnormal position. She had a history of two prior vaginal deliveries and bilateral tubal ligation 10 years ago. 

On further investigations, an ultrasonographic examination showed the presence of a sizable intramural uterine fibroid that measured roughly 20 cm by 15 cm which was the cause of the uterine growth. Due to the chronic menorrhagia, hemoglobin levels significantly dropped. The lab investigations of the patient are depicted in Table 1.

**Table 1:** Clinical findings of the blood investigation C-reactive protein (CRP), mL (millilitre), g/dL (gram per decilitre), mg/dL (milligram per decilitre), mEq/L (milliequivalents per litre), mmol/L (millimoles per litre), µL (microlitre)

Serum parameters	Levels recorded	Normal range
White blood cell count	12.4 x 10^5^/mL	4.0-11.0 x 10^5^/mL
Haemoglobin	7.3 g/dL	12.0-17.0 g/dL
C-reactive protein (CRP)	9 mg/dL	0.3-1.0 mg/dL
Na^+^	119 mEq/L	135-145 mEq/L
K^+^	3.7 mmol/L	3.5-5-5 mmol/L
Lactate	1.7 mmol/L	0.5 – 2.0 mmol/L
Platelet	2.5 x 10^5 ^/µL	1.5-3.0 x 10^5^/µL

The patient was advised on treatment possibilities, such as myomectomy and hysterectomy. After careful consideration, she decided to take a decisive action and underwent an abdominal hysterectomy given the sizeable fibroid and accompanying symptoms. Before planning the hysterectomy, the patient was taken up for uterine artery embolization to minimize the blood loss during surgery and MRI was done to map the involvement of soft tissues around the tumor. Uterine artery embolization was done using polyvinyl alcohol crystal two days prior to hysterectomy in the interventional radiology department. This procedure helped the surgeons to perform the surgery with minimum blood loss, since the woman was already anemic.

**Figure 2:**
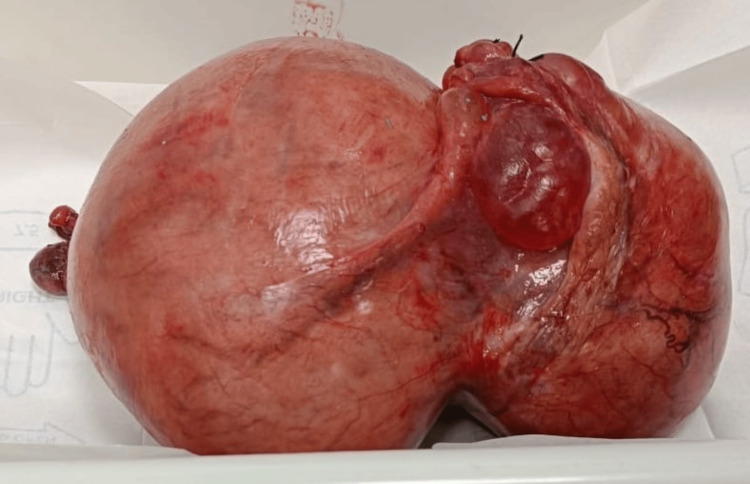
Resected uterine fibroid

**Figure 3:**
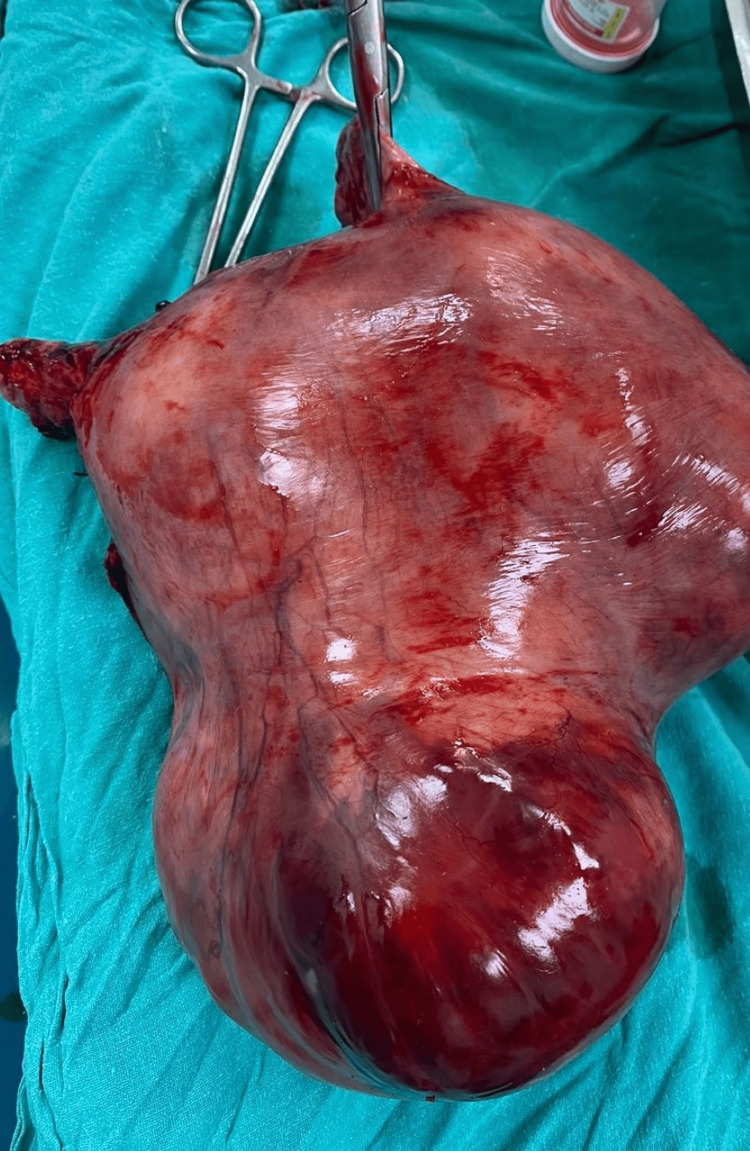
Uterine fibroid - longitudinal aspect

An abdominal midline incision was done while the patient was under general anesthesia to allow access to the abdominal cavity. The uterus, which had become noticeably larger and included a sizable intramural fibroid, was painstakingly resected which weighed around two and a half kilograms as depicted in Figure 2 and Figure 3 respectively. The abdominal wall was then stitched up keeping approximation of all the layers, and the uterus and fibroid were removed.

**Figure 4:**
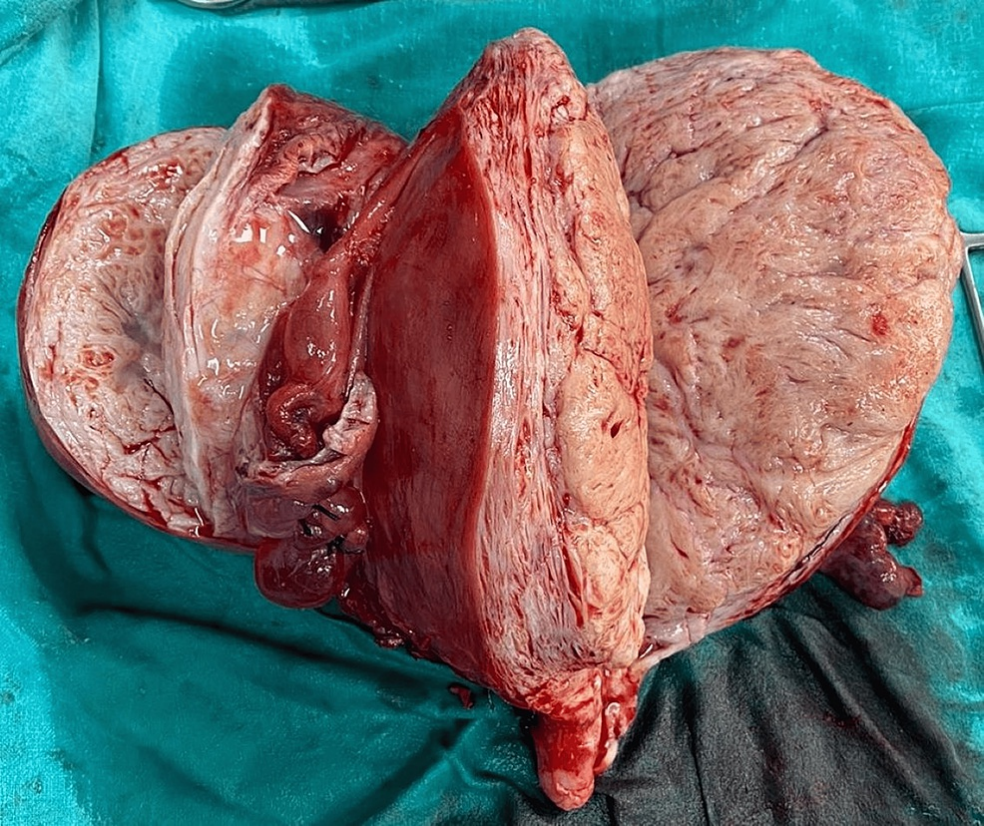
Cut section of the resected fibroid

Cut section of the fibroid was examined to check for the possibility of any lesions. On gross examination the mass was pink in color with no signs of necrotic tissues or hemorrhage which was well circumscribed and homogenous in texture. The cut section of the lesion is shown in Figure 4.

**Figure 5:**
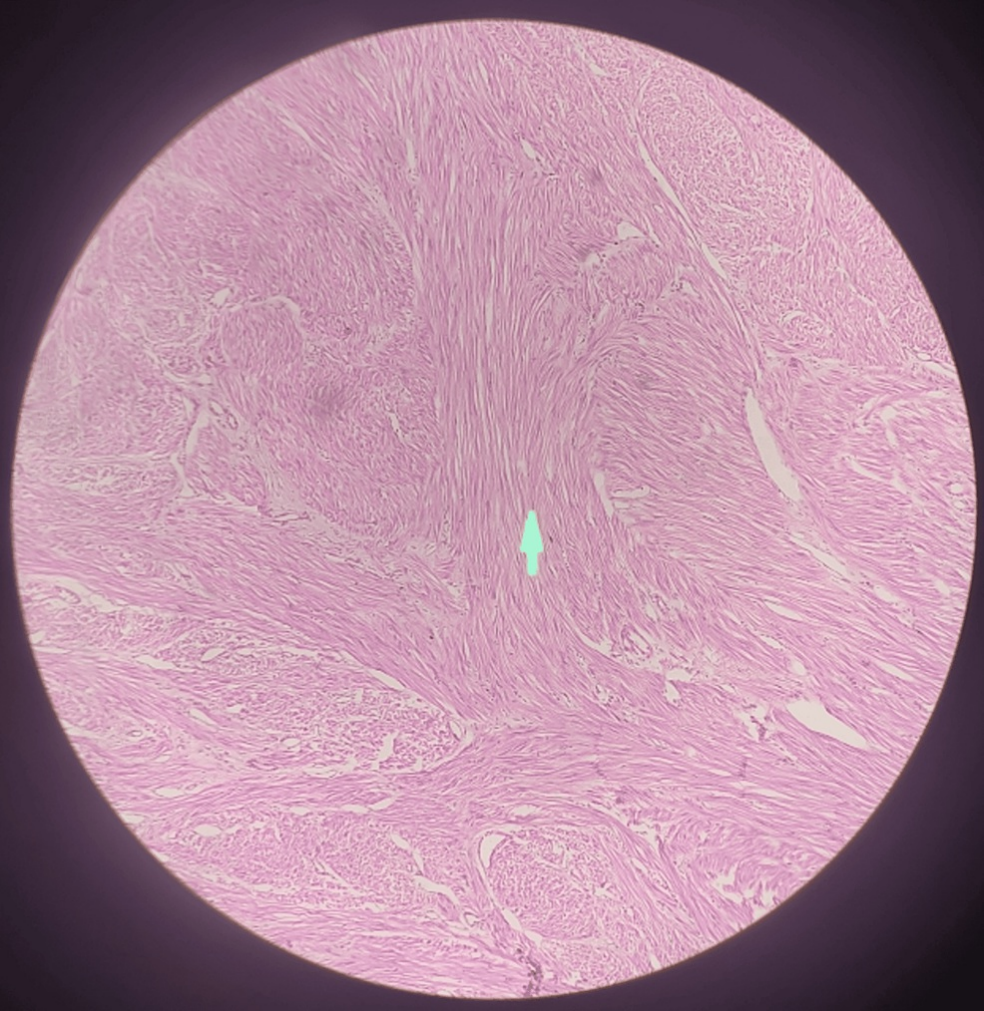
Histopathological slide of the leiomyoma The arrow depicts the spindle-shaped smooth muscle cells

The cut section of the specimen was sent for histopathological examination, that revealed a leiomyoma as suspected. The slide shows bundles of spindle-shaped smooth muscle cells and a whorled or swirling pattern of growth, in Figure 5. 

The intra-operative blood loss was around 100-120 milliliters, which is permissible given the anemic condition of the woman. The patient's surgical recovery went without incident. Following surgery, her menorrhagia and abdominal pain symptoms subsided, and hemoglobin levels improved. On the fifth postoperative day, she was sent home from the hospital with instructions for aftercare. 

## Discussion

Twenty to 40% of women of reproductive age suffer from uterine fibroids [5]. Advanced age, early menarche, family history, obesity and nulliparity are a few factors that increase the chances of fibroids. According to a recent survey in 2018, 17 women in every 1000 married women undergo hysterectomy every year in India because of fibroids [6,7]. Fibroids are benign tumors that develop from the myometrium, or smooth muscle tissue of the uterus. Estrogen and progesterone are necessary for the creation of fibroids [8]. Before puberty, fibroids are uncommon, they become more common during periods of reproduction, and they get smaller after menopause. Estradiol is produced endogenously in fibroid tissue by aromatase, and fibroid stem cells express estrogen and progesterone receptors, which promote tumor growth when these hormones are present [9]. Based on location, uterine fibroids are classified as intramural (inside the myometrium), sub-serosal (protruding from the uterus) and submucosal (bulging into the uterine cavity) [10]. The number, size and location are influencing factors for treatment and symptoms of the fibroids [2,11]. Likewise, due to the extensive size of the fibroid in the mentioned patient, the only treatment options were either myectomy or hysterectomy. 

The initial part of investigation of fibroids is ultrasonography which gives reliable evidence. Other methods include transvaginal ultrasonography which is susceptible to detecting uterine fibroids 90-99% but it has a small drawback where it fails to detect sub-serosal fibroids [12]. Uterine artery embolization is a tried pre-operative procedure that prevents excessive blood loss during surgery. This procedure is advised for suitable patients who fulfill the criteria of being a pre-menopausal woman, history of heavy menstruation and having no desire for pregnancy [13]. Management of such fibroids depends greatly on size and location, in this case the complexities associated with managing giant uterine fibroids, particularly in a rural tertiary care setting, were rather challenging. The woman went through a number of years without the fibroids being diagnosed and hence the size of the fibroids increased to this extent. This is usually due to lack of knowledge and awareness along with decreased care facilities in primary health care centers.

Two types of treatment therapies are there that can be used in case of fibroids: medical and surgical. Medical therapy is only effective for fibroids that are small in size or it can be used as a preoperative treatment to reduce the size of the tumor before surgery in peri-menopausal or menopausal women. Drugs used are oral contraceptives, tranexamic acid, levonogestrel-releasing intrauterine system (Mirena) [14]. In cases where fibroid dimensions and symptoms pose substantial health risks, as evidenced here, an abdominal hysterectomy can serve as a definitive therapeutic modality.

## Conclusions

The article reflects well upon both the successful management of the patient and the importance of an individualized, multidisciplinary approach involving an anesthetist and interventional radiologist to address the specific needs of the patient. In this case a 45-year-old woman with a single large fibroid developed symptoms such as heavy menstruation, pressure symptoms and pelvic pain. The diagnosis was confirmed by imaging studies which helped to plan and consider treatment options. This case report highlights the importance of individualized care and shared decision-making in the management of uterine fibroids. By careful consideration of the patient's needs and optimal recovery, an abdominal hysterectomy was executed successfully, resulting in symptom relief and an improved quality of life for the patient.
